# Supplementary figures and images for: Epac2 in midbrain dopamine neurons contributes to cocaine reinforcement via enhancement of dopamine release
Source: eLife. 2022 Aug 22;11:e80747. doi: 10.7554/eLife.80747 (PMC9436413; doi:10.7554/eLife.80747)

WT mice

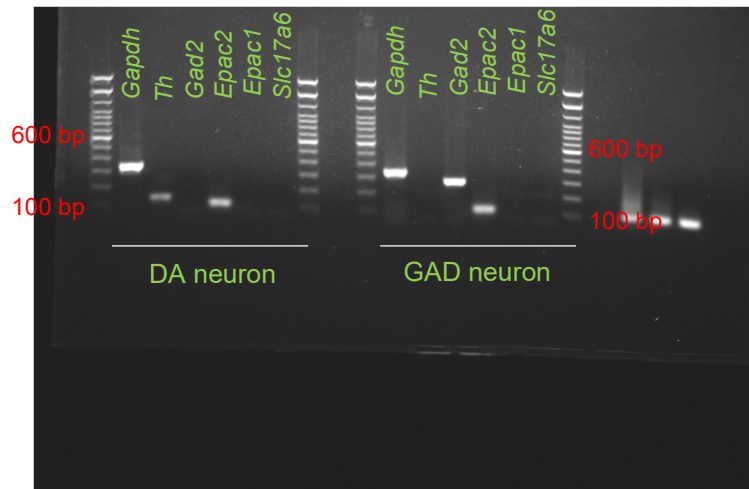

Epac2-cKO mice

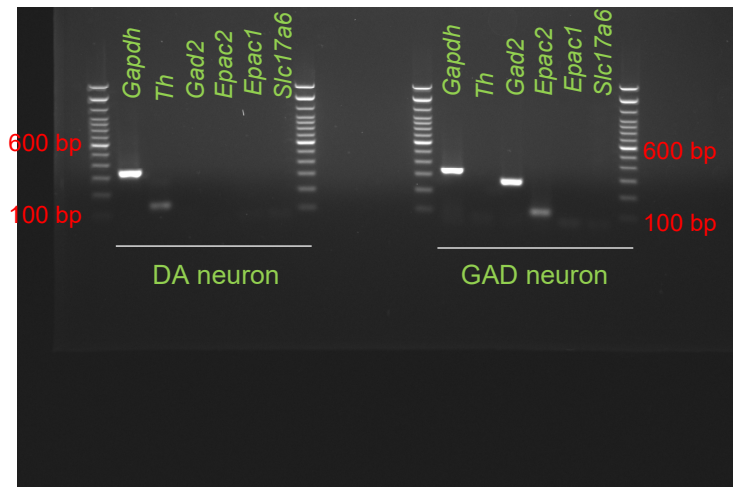

Supplement: Figure 2—figure supplement 2—source data 1. [file elife-80747-fig2-figsupp2-data1.zip › Figure 2 - figure supplement 2 - source data 1/Figure 2-figure supplement 2-source data1.pdf]
